# Time-dependent complexity characterisation of activity patterns in patients with Chronic Fatigue Syndrome

**DOI:** 10.1186/s13030-024-00305-9

**Published:** 2024-04-02

**Authors:** Paloma Rabaey, Peter Decat, Stefan Heytens, Dirk Vogelaers, An Mariman, Thomas Demeester

**Affiliations:** 1 IDLab, Department of Information Technology, Ghent University - imec, Ghent, Belgium; 2 Department of Public Health and Primary Care, Ghent University, Ghent, Belgium; 3 Center of Integrative Medicine, Department of Physical Medicine and Rehabilitation, University Hospital Ghent, Ghent, Belgium; 4 Department of General Internal Medicine, AZ Delta, Roeselare, Belgium

**Keywords:** Chronic Fatigue Syndrome, Complex adaptive systems, Activity patterns, Time-dependent complexity, Fractal dimension, Personalised monitoring

## Abstract

**Background:**

Chronic Fatigue Syndrome patients suffer from symptoms that cannot be explained by a single underlying biological cause. It is sometimes claimed that these symptoms are a manifestation of a disrupted autonomic nervous system. Prior works studying this claim from the complex adaptive systems perspective, have observed a lower average complexity of physical activity patterns in chronic fatigue syndrome patients compared to healthy controls. To further study the robustness of such methods, we investigate the within-patient changes in complexity of activity over time. Furthermore, we explore how these changes might be related to changes in patient functioning.

**Methods:**

We propose an extension of the allometric aggregation method, which characterises the complexity of a physiological signal by quantifying the evolution of its fractal dimension. We use it to investigate the temporal variations in within-patient complexity. To this end, physical activity patterns of 7 patients diagnosed with chronic fatigue syndrome were recorded over a period of 3 weeks. These recordings are accompanied by physicians’ judgements in terms of the patients’ weekly functioning.

**Results:**

We report significant within-patient variations in complexity over time. The obtained metrics are shown to depend on the range of timescales for which these are evaluated. We were unable to establish a consistent link between complexity and functioning on a week-by-week basis for the majority of the patients.

**Conclusions:**

The considerable within-patient variations of the fractal dimension across scales and time force us to question the utility of previous studies that characterise long-term activity signals using a single static complexity metric. The complexity of a Chronic Fatigue Syndrome patient’s physical activity signal does not suffice to characterise their high-level functioning over time and has limited potential as an objective monitoring metric by itself.

## Background

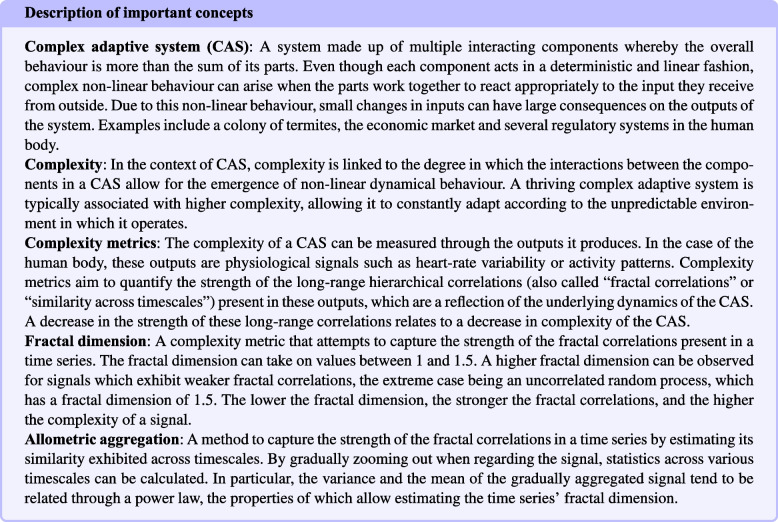



### The human body as a complex adaptive system

The traditional reductionist approach to Western medicine focuses on specific biological causes of a disease, and goes on to treat these causes in order to alleviate symptoms [1]. This approach fails when the complex interactions between many parts and mechanisms within the human body lie at the root of the illness, making it impossible to isolate a single failing part which can be treated. This seems to be the case for several chronic and functional syndromes such as Chronic Fatigue Syndrome (CFS) or fibromyalgia [2]. In this case, it might be more beneficial to take on a complex adaptive systems (CAS) perspective while studying such phenomena. This perspective treats the human body as being made up of several components, constantly interacting with one another and reacting to external influences, allowing for complex behaviour to emerge [3]. While all the components might act in a deterministic and linear fashion, the total behaviour of the system can be more than the sum of its parts. In this context, health can be seen as a complex system that arises from hierarchical network interactions between a person’s external environment and internal physiology, as stated by Sturmberg et al. [4].

The complexity and underlying dynamics of regulatory systems in the body can be quantified through the outputs they produce [5]. Physiological output signals, such as heart-rate variability (HRV) or activity patterns, exhibit a measure of self-similarity that is reflective of the complex feedback loops present in the system which created them [6]. This self-similarity can be measured through the amount of long-range correlations which are present in the signal. For example, current values of the heart-rate variability signal can be related to values which will arise in the distant future, since the emergent properties of the system allow small effects to carry large consequences [7]. Various methods have been developed to capture and quantify the strength of such correlations [6], including allometric aggregation [8], which we will address in detail in the Methods section. The hierarchical patterns that are formed through these long-range correlations are fractal-like, meaning they repeat themselves across several timescales [9]. This is why the term “fractal complexity” can be used to describe the strength of self-similarity found in these signals.

Complexity analysis of these outputs has shown potential to capture changes in health status of individuals, both regarding specific syndromes and more general processes like ageing. For example, a decrease in long-range correlations in the HRV signal (which can be interpreted as a decrease in complexity of the underlying regulatory system) might indicate a patient at risk of cardiac arrest [10], and a decrease in fractal complexity of activity patterns can be related to mortality risk in (older) adults [11].

### Chronic Fatigue Syndrome as a disruption of the CAS

The possibility to view a disease from a CAS point of view is especially welcome in situations where the classical cause-effect view fails to explain the symptoms of a patient. This is the case for the Chronic Fatigue Syndrome, where it has continuously proven difficult to pinpoint a single pathophysiological cause responsible for the symptoms experienced by these patients [12]. For this reason, diagnosis of the syndrome is a process of elimination [13], made even more difficult by the fact that there seems to be a partial overlap in symptom manifestation with other similar syndromes, such as fibromyalgia [14]. The term Bodily Distress Syndrome (BDS) has been proposed as an umbrella term to capture a range of syndromes which are poorly defined due to a lack of medical causes of the observed symptoms [15]. This suggests that syndromes like CFS and fibromyalgia might be different symptom manifestations of a disruption in the underlying CAS [16], more specifically the autonomic nervous system [2] or hypothalamic-pituitary-adrenal axis [17]. This degraded performance of the CAS hinders the patient from adequately responding to unpredictable stimuli and stresses, resulting in symptoms such as fatigue and pain.

Viewing CFS and similar syndromes as a disruption of the patient’s CAS is reminiscent of taking on a biopsychosocial approach, which allows for the inclusion of psychological and social aspects in the study of how a disease manifests itself in a particular patient [18]. While these approaches indeed complement each other well, especially in the context of patient-centred care, they are not one and the same: the CAS point of view we take on here also allows us to *quantify* the extent of the disruption in a patient’s CAS. Comparing the fractal properties of CFS patients’ HRV signals to those of healthy subjects indeed confirmed a reduced complexity in the CFS patient group [19]. Additionally, CFS patients’ long-term activity signals (recorded over 12 days) also showed a decreased complexity (measured through the method of *allometric aggregation*) compared to controls, though the significance of this result depends on the average activity level of the studied group [20, 21]. Nevertheless, both studies identify physical activity as a possible indicator of the complexity of the underlying CAS of a CFS patient.

The presented applications of the CAS perspective might open up possibilities for new diagnostic methods for CFS, though robustness of the complexity metrics needs to be studied further before applying them to this end. In this study, we dive deeper into the use of activity patterns to quantify complexity, choosing allometric aggregation as a complexity method to allow comparison with Burton et al. [21]. One aspect which has not been studied is how the complexity of a physiological signal may change over time, and what this implies about the validity of using a single static complexity to characterise a long-term activity signal. Where previous studies merely focused on using complexity to compare across patients and controls for diagnostic purposes, we focus on studying the within-patient evolution of the complexity over time.

While diagnosis of CFS is clearly not an easy process, treatment of the syndrome is certainly difficult as well [22], leaving most patients functionally impaired for several years [13]. Little is known about what triggers these patients to enter prolonged periods of dysfunctioning, and as a result anticipation and mitigation of these situations is difficult. By viewing the patient as a complex system in which certain internal and external triggers can have unexpected consequences in regards to patient functioning, new insights could be obtained in how to prevent such situations, ultimately contributing to the creation of novel treatment methods. To this end, a time-dependent complexity characterisation of activity patterns could be a way to quantify changes in the properties of a patient’s complex system. If personal changes in patient functioning can be linked to changes in complexity, the temporal evolution of the complexity could be used to monitor and track the underlying disease state of a particular CFS patient. This constitutes another reason to further explore the potential of the allometric aggregation method and develop its extension over time. In this context, we investigate the following hypothesis: *periods of decreased functioning for a particular patient are coupled with a lower complexity of the patient’s activity pattern.*

The motivation for the choice to study activity patterns in particular is two-fold. On the one hand, previous research has shown the potential of patient activity to reveal information on the mechanisms which drive the perpetuation of CFS [23, 24], and a complexity analysis of these signals seems to be meaningful [20, 21]. Secondly, accelerometry devices make it possible to reliably capture long-term continuous activity recordings from subjects in free-living conditions at a low cost [25].

### The need for personalised treatment

Due to the heterogeneous nature of CFS as a syndrome, it can be said that all CFS patients are diseased in their own way [12, 26]. While cognitive behavioural therapy has shown evidence of clinical improvement in some patients, it turns out ineffective in others [27]. The heterogeneity of the syndrome clearly calls for individually tailored treatment strategies [22], but a systematic approach on how to obtain these is currently lacking. To this end, there is a need for techniques which can identify patient-specific perpetuating factors [28]. This fits in with a more general trend within psychosomatic research, which constitutes a move from studying between-person associations for a given diagnosis to identifying within-patient dynamics [29]. Note that the personalised treatment approach aligns well with the CAS point of view on CFS: each patient is governed by their own complex system, which is bound to react to triggers that are dependent on their (medical) history and specific surroundings, and which are therefore not necessarily shared among patients. For this reason, special attention will be paid to the potential of a time-dependent complexity method to quantify these individual changes in a patient’s complex system, while limiting comparison across patients.

### Our contributions

In short, our contributions can be summed up as follows:

- We describe an extension of the allometric aggregation method which can capture temporal changes in complexity of a time series. Its properties are demonstrated through activity patterns which are obtained from CFS patients, though its application is not necessarily confined to this use-case alone.
- Using our new method, we demonstrate the extent of within-patient temporal variations in the complexity of their activity patterns, showing that the complexity can be highly non-static. In several of our patients, the changes in complexity over time were larger than the difference in complexity measured between patient and control groups in a previous study by Burton et al. [21]. This raises questions about the utility of using a single static complexity value inferred from a long-term activity signal for diagnostic purposes, and forms a possible explanation for the limited power of the associations they report.^1^
- The possible link between personal variations in functioning and the complexity of a patient’s activity pattern is investigated. More specifically, the following hypothesis is explored: *weeks during which a particular patient showed decreased functioning are coupled with a lower complexity of that week’s activity pattern.* Finding no consistent relation between functioning and complexity, even on a patient-by-patient basis, we currently cannot confirm this hypothesis.
- A novel data set is published, which aligns continuous activity recordings obtained from multiple CFS patients with daily indications of symptom severity and other indicators of general functioning. As well as forming the basis for the results presented in this work, these 3-week measurements can be used in future research to explore patient-specific triggers for dysfunctioning and their relation to physical activity or derived metrics. The data and code to reproduce the reported complexity analysis is available at our Github repository [30].

## Methods

### Data collection

Longitudinal data was gathered from 7 CFS patients (6 female, 1 male) over a period of 3 weeks (20 or 21 days), following an Ecological Momentary Assessment (EMA) study design [31]. The patients were recruited through their involvement in the CFS treatment track at Ghent University Hospital (Belgium). Apart from having received a CFS diagnosis according to the Fukuda criteria [13], the only other criterion for inclusion was the patient’s willingness to participate in the 3-week follow-up period. The small number of patients can be justified by the fact that the focus is on studying within-patient variations in complexity and functioning. Generalisations across patients are not the objective of the current research. Instead we aim to present a complete picture of how complexity methods can be applied to obtain a time-dependent characterisation of patient conditions.

In order to track the activity patterns of the patients, the Axivity AX3 [32] device was used. This device allows to record raw accelerations across 3 orthogonal axes, at a sampling frequency of 50 Hz. Subjects were asked to wear the monitor on their non-dominant wrist continuously for the 3-week observation period. The decision to go for a wrist placement (rather than hip or ankle) was made with ease-of-use and compliance in mind, benefiting the continuity of the recorded activity signal. Manual inspection of the data confirmed that instances of non-wear time were sparse and of short duration. All activity signals were therefore deemed reliable enough in terms of continuity. For every patient, the fine-grained raw accelerations were summarised into a sequence of activity counts. This was done in order to allow comparison of our results with previous work conducted by Burton et al. [21], as will be elaborated on in the Discussion. These counts can be interpreted as a score representing the intensity of activity during each 1-minute period of the recording.^2^ The process used to convert raw accelerations into activity counts can be found in the supplementary material (see [30]) and is roughly based on the method described by Brønd et al. [33].

At the end of every day during the recording period, participants were asked to fill in an online questionnaire regarding their functioning throughout that past day. Reminders to fill in this survey were sent out each day at 8:00 p.m. via email. The survey contained questions on mental and physical well-being of patients, questioned separately for the morning, afternoon and evening. Patients were also asked to indicate their main activity for each of those day segments, and they were questioned on their opportunities to relax and recharge throughout the day. The surveys were somewhat personalised to accommodate each patient, but the core structure remained the same in each case. More information on the exact contents of the surveys can be found in the supplementary material (see [30]). In the current study, we did not use any of the indicators gathered through this daily survey, as we wanted to focus most on studying the temporal variations in complexity of the activity signals. However, we believe that these (sub-)daily indicators of patient symptoms and well-being show much potential for future CFS research, which is why we publish the full dataset along with our current findings.

To get an idea of the patients’ high-level functioning throughout the observation period, each patient was followed up closely by a general-practitioner-in-training. They conducted weekly in-depth interviews with the patient, which were partially guided by the information obtained through the daily survey for that past week. Based on these interviews, the physicians ranked the three weeks in the follow-up period in terms of the patient’s general high-level functioning during those weeks. This allows us to investigate whether there are indications of an alignment between patient functioning and the complexity of their activity sequence. The decision to settle on a ranking rather than assigning absolute levels of functioning to each week, was two-fold. On one hand, each general-practitioner-in-training might have a different interpretation of such absolute levels of functioning. Secondly, a relative ranking of the weeks allows relative comparison of within-patient functioning over time. This suffices in our case, as we don’t aim to compare across patients. Note that in some cases, the physician was only able to point out the worst or best week in terms of functioning, resulting in a partial ranking.

We would like to point out the potential of this data set for future research. Information was gathered on three levels: (1) the recorded accelerations, corresponding to low-level, high-granularity information on a patient’s activity, (2) the surveys, capturing daily indications on several mental and physical features, and (3) the in-depth interviews, finally contextualising the previous levels using qualitative information. This makes for a rich data set, which can form the basis for further exploration of the link between perceived functioning and objective physical activity. Only a part of the information it holds was exploited for the research presented here.

### Quantification of complexity

As a method to quantify the complexity of a time series, we choose allometric aggregation. One reason for this choice of metric is that it has been used in a past study to measure the complexity of activity patterns [21], showing (to some extent) reduced complexity in CFS patients compared to healthy controls. Compared to other methods to capture long-range correlations [6], it is relatively straightforward to calculate the fractal dimension using allometric aggregation, making it a good candidate for extension over time.

The allometric aggregation method captures a time series’ similarity across timescales by identifying correlations across these scales and calculating the so-called fractal dimension of the signal [8]. Intuitively, this means zooming out further and further (by aggregating the samples in the series) to analyse the time series statistics on these scales. The fractal dimension can be seen as a measure of the information needed to describe a system across different scales. When correlations across scales are higher, the amount of information needed to describe them is lower, and the fractal dimension will be lower as well. An uncorrelated random process will have a fractal dimension of 1.5, while a fully regular process will have a fractal dimension of 1. Healthy processes in the human body should operate somewhere in between these two extremes, with more complex processes straying further away from the fractal dimension of an uncorrelated random process. For this reason, lower fractal dimensions are seen as an indication of a system which exhibits higher complexity.

The allometric aggregation method, as first described by West [8], is defined as follows. As an input, the algorithm takes the full time series $T = \{y_i\}_{i=1,...,N}$ consisting of *N* consecutive and equidistant samples. These samples are aggregated by grouping them into *K* non-overlapping blocks of size *n*, as illustrated in Eq. (1). To ease the representation, it is assumed that *T* can indeed be divided into exactly *K* blocks of size *n*. Equation (2) shows how a new representation of the series, $Y^{(n)}$, is obtained by taking the sum within each block. By including more samples in a block, a higher level of aggregation is considered, which corresponds to viewing the series on a larger timescale. The aggregation process is repeated for increasing block sizes *n*, with $n = 1...n_{max}$. The parameter $n_{max}$ decides the maximum scale on which the strength of the correlations between the samples in the series will be evaluated.

1 $$\begin{aligned} T = \{\underbrace{y_1,...,y_n}_\text {1}; \underbrace{y_{n+1},...,y_{2n}}_\text {2};...; \underbrace{y_{(K-1)n+1},...,y_{Kn}}_\text {K}\} \end{aligned}$$

2 $$\begin{aligned} Y^{(n)} = \{Y_1^{(n)},...,Y_K^{(n)}\},\ \text {with}\;Y_k^{(n)} = \sum _{i=1}^{n} y_{(k-1)n+i} \end{aligned}$$

At every scale, i.e. for every considered value of the aggregation level *n*, the mean $\overline{Y}^{(n)}$ and variance $var Y^{(n)}$ of the series are recorded on a log-log chart. If a straight line can be fitted through the plot, the mean and variance are related through a power-law relation, as shown in Eq. (3). When this power law is present, the signal is said to have fractal properties (up to the maximum scale $n_{max}$). The fractal dimension *D* can be calculated through the parameter *b*, representing the slope of the relation on the log-log plot, as $D = 2 - b/2$ [34]. A practical implementation of the method we just described is provided by Algorithm 1 in the Appendix.

3 $$\begin{aligned} var Y^{(n)} = a\cdot (\overline{Y}^{(n)})^{b} \end{aligned}$$

### Adapted allometric aggregation (AAA)

In the allometric aggregation method (as shown in Algorithm 1), one must set a value for the parameter $n_{max}$, which decides the range of scales over which to evaluate the strength of the fractal correlations. When investigating this original method, we found that the fractal dimension can vary considerably depending on the choice of $n_{max}$. Fitting a single straight line to the log-log plot does not always capture how the slope of the relation changes as scales grow bigger (support for this claim will be provided in the Results section). Instead of running the algorithm multiple times with various choices of $n_{max}$ in order to explore the dependence of the outcome on this parameter, a simple change is made to the original method. By fitting a third-order polynomial along the logarithmic relation between the mean and the variance, rather than one straight line, a local slope can be obtained for any value of the aggregation level *n* through derivation. An order of three is chosen since it ensures enough flexibility in capturing the change of the slope across scales, while still being sufficiently robust.

We also advocate for calculating the required slope from a characteristic that is evenly spread in log-log space over the aggregation levels *n*, rather than clustered towards the highest range of *n*. Instead of *incrementing* *n* by 1 after every aggregation step, we therefore *multiply* *n* with a constant factor $s > 1$.

Preliminary analysis of the first version of the method revealed that its outcome is unreliable when the block size *n* is relatively large compared to the full length of the considered sequence, due to the low number of aggregated blocks from which the mean and variance have to be estimated in that case. To address this issue, another change can be made: instead of considering non-overlapping blocks of samples (cf. Eq. (1)), allowing some overlap of the blocks effectively increases the number of segments from which the mean and the variance are estimated in a particular aggregation step.

Algorithm 2 in the Appendix presents a practical implementation of the adapted version of the allometric aggregation method as described in this section. From now on, we will refer to this algorithm as the AAA method (for *adapted allometric aggregation*).

### Temporal adapted allometric aggregation (t-AAA)

The allometric aggregation method has previously been used to obtain fractal dimensions for 12-day activity signals [21] and for 6-8 minute HRV signals [34]. In both cases, the method was applied to the physiological signals in their entirety, resulting in a single value for the fractal dimension representing the overall complexity of the signal. This makes less sense in the former application than in the latter, as one can imagine that the underlying complexity reflected by an activity signal recorded over multiple weeks might not be as static as this single complexity value suggests.

**Fig. 1:**
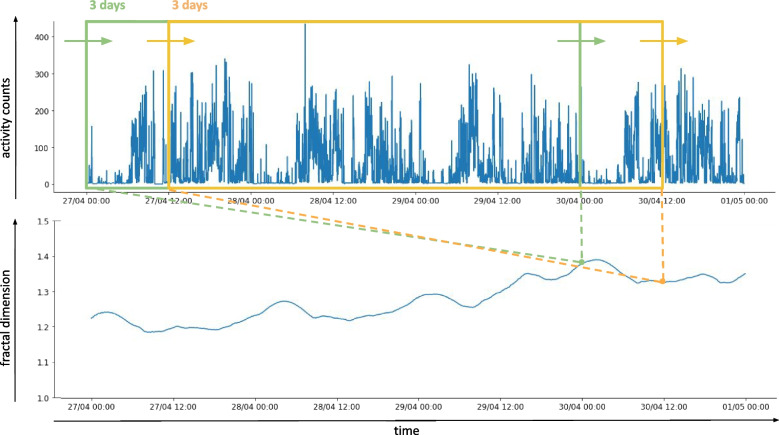
Time-dependent complexity. Illustration of the main idea for capturing the evolution of the complexity over time. A sliding window, as depicted here with a width of 3 days (72 hours), advances along the time series, in this case an activity counts sequence. Within the window, the AAA method is applied to obtain the fractal dimension. These dimensions form a new signal, quantifying the evolution of the complexity of the activity signal over time

For this reason, the AAA method as described above is extended to obtain an evolution of the complexity over time. Instead of feeding the entire long-term sequence into the method at once, the algorithm is applied in a windowed fashion, as is presented conceptually in Fig. 1. As the window slides across the sequence, the fractal dimension is calculated based only on the sequence of datapoints contained within that particular window. The resulting fractal dimensions form a new sequence reflecting the evolution of the complexity over time. The subsequent windows may overlap, and the steps with which the window advances decide the granularity of the obtained complexity signal. The width of the window should be chosen large enough so that enough data is available for a reliable calculation of the fractal dimensions. At the same time, setting a wider window limits the temporal resolution of the resulting time-dependent complexity signal.

In short, the proposed time-dependent complexity method extracts a two-dimensional complexity signal from the activity sequence: the first dimension signifies the changes over time, and the second captures subtle changes in the self-similarity across scales. The final time-dependent method, which consists of consecutive applications of the AAA method using a sliding window, is implemented by Algorithm 3. From now on, we refer to this method by t-AAA, for *temporal adapted allometric aggregation*.

## Results

In this section, we apply the adapted allometric aggregation method to the activity sequences and demonstrate some of its properties. First, the method is applied to the full 3-week activity sequence of a randomly selected patient, to illustrate how the fractal dimension may change depending on the scaling parameter *n*. Next, we compare weekly measures of the fractal dimension to the overall dimension obtained for the full 3-week activity sequences, to show that complexity is not a static feature. We then investigate the relation between weekly complexity and global functioning for each patient. To further explore the variations in complexity over time, we apply our newly devised time-dependent method to obtain an evolution of the complexity for each CFS patient. Finally, correlations between the obtained complexity signal and the activity sequence from which it was extracted are reported for every patient, as well as some general properties of each patient’s activity pattern.

### Variations in complexity across scales

**Fig. 2:**
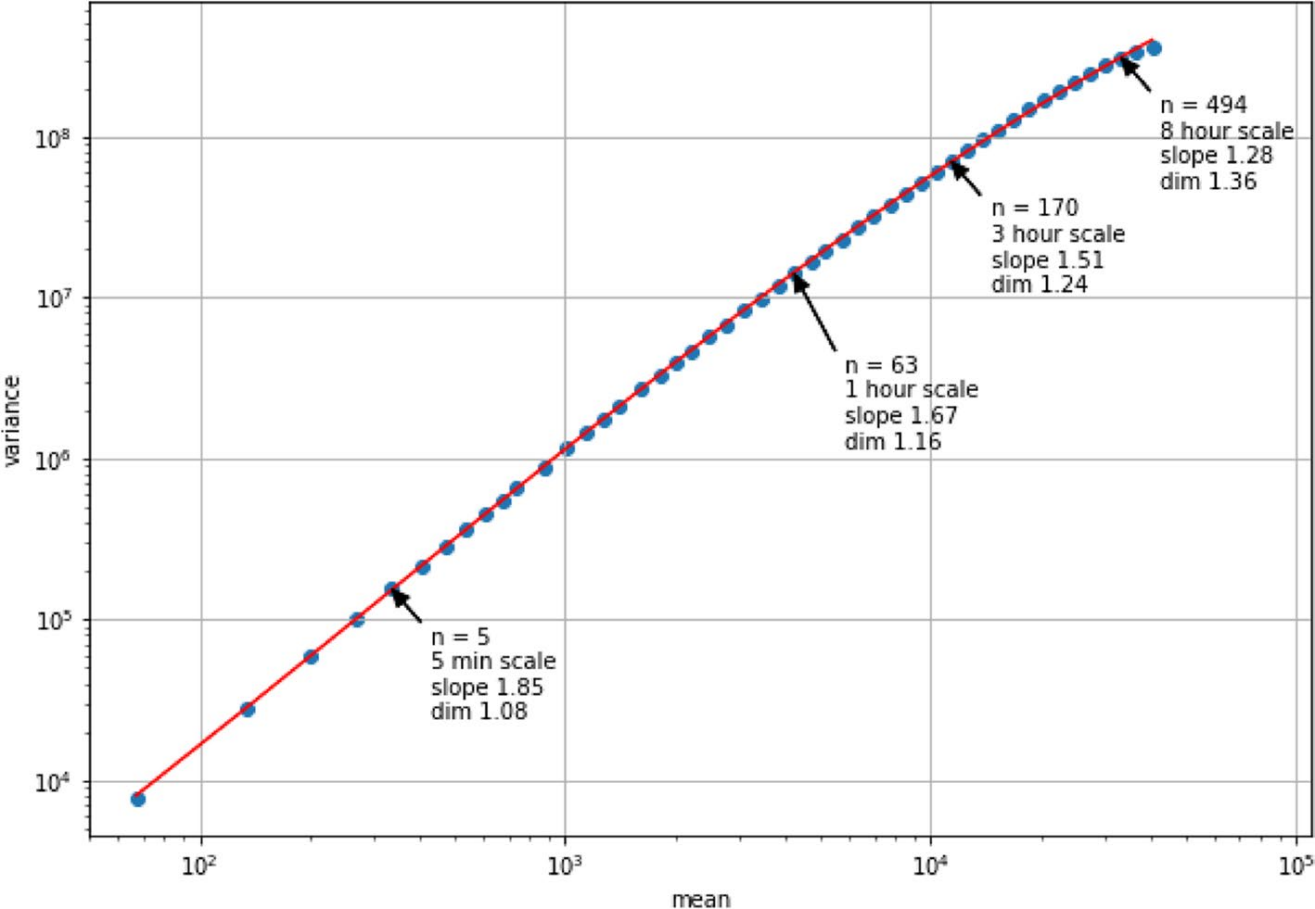
Illustration of scaling behaviour for a randomly selected patient. Fitting a polynomial to the log-log relation between the mean and variance for different levels of aggregation allows to determine a slope for any value of n. This in turn can be converted to the fractal dimension of the activity sequence around that particular range of scales. To obtain these results, the AAA method was applied to the full 3-week activity sequence of a patient chosen at random, with $n_{min}=1$, $n_{max}=10\times 60$ and $s=1.1$

First, we present some insights into the meaning of the time scale at which the complexity analysis is performed (more specifically, parameters *n* and $n_{max}$ in the AAA method). To this end, we study the output of the AAA algorithm when the full 3-week activity sequences are used as an input, rather than immediately moving on to the time-dependent characterisation of their complexity. Figure 2 shows the log-log plot which is obtained when the AAA method is applied to the full activity sequence of a patient chosen at random (with parameters $n_{min}=1$, $n_{max}=10\times 60$ and $s=1.1$). The continuous line represents the third-order polynomial which is fitted to the relation between the mean and the variance. As addressed in Eqs. (1) and (2), the algorithm aggregates the counts into blocks of size *n*, for which the mean and variance are obtained and compared on the log-log plot. In Fig. 2, some of these datapoints are annotated with the aggregation level *n* for which they were obtained. For any such point on the plot, the local slope of the mean-variance relation can be calculated and converted into the local fractal dimension for that particular aggregation level, as is depicted in the figure as well.

Conceptually, these values of *n* can be seen as the scale up to which we zoom out to view the time series. Since we know that $n = 1$ corresponds to one sample, which represents a 1-minute interval in the patient’s activity recording, *n* can easily be converted to a timescale, which is also annotated on the plot. For example, when $n=170$, the samples in the activity series are aggregated up to a scale of around 3 hours. Intuitively, this comes down to studying the variations in activity exhibited by all 3-hour segments which are contained in the 3-week recording. When there is a stronger change in variations as we move from one aggregation level to the next, the slope obtained around this timescale will be steeper, resulting in a lower fractal dimension (which is associated with higher complexity).

**Fig. 3:**
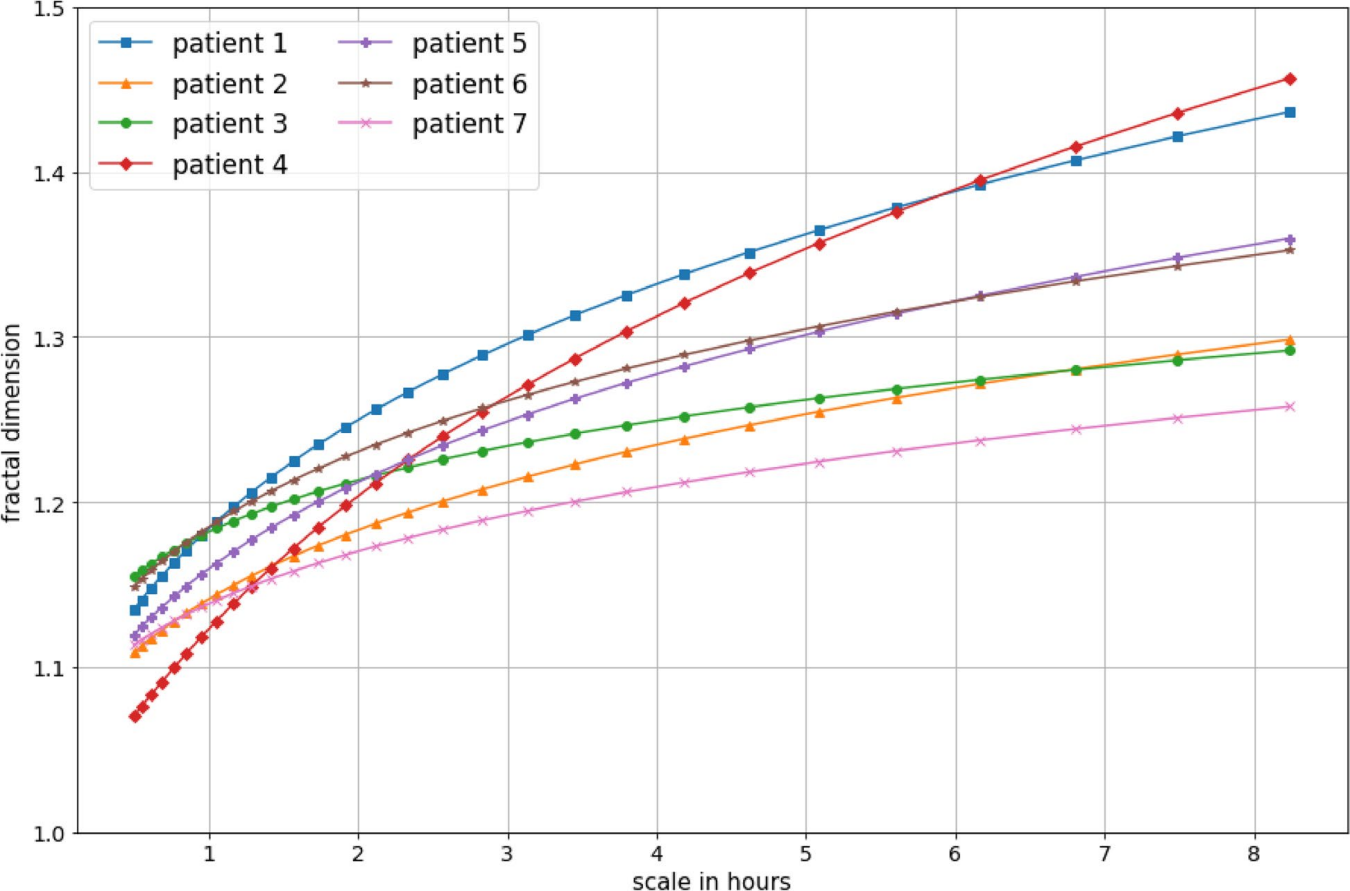
Variations in complexity across scales. The fractal dimension which is obtained for a range of scales is depicted per patient. To obtain these dimensions, the AAA method was applied to the full 3-week activity sequence for all patients (with $n_{min}=1$, $n_{max}=10\times 60$ and $s=1.1$) and the slope of the mean-variance relation was evaluated for various aggregation scales *n* lying between $n_{min}$ and $n_{max}$

From Fig. 2, we can learn that higher fractal dimensions are estimated for higher timescales. To show that this is the case for all patients, the AAA method is applied to the full activity sequences, again with parameters $n_{min}=1$, $n_{max}=10\times 60$ and $s=1.1$. Figure 3 shows the fractal dimensions which are obtained for scales ranging from 30 minutes to around 8 hours. While the relation between scale and complexity follows a similar trajectory for each patient, it is clear that relative comparisons of the fractal dimension between patients are dependent on the scale. For example, while patient 4’s activity pattern shows the highest complexity for the 30-minute scale, it shows the lowest out of all patients for the 8-hour scale.

### Variations in complexity over time

Table 1 reports the static fractal dimensions and activity sequence properties per patient. All fractal dimensions listed in the table were obtained by applying the AAA method with $n_{max}=9\times 60$ (corresponding to a maximum scale of 9 hours) and evaluating the slope at $n=3\times 60$ (corresponding to a scale of 3 hours). The global fractal dimension is obtained by using the full 3-week activity sequences as an input to the AAA method. The weekly values are the result of applying the same algorithm to the subsequences recorded from day 1 to day 7 (week 1), day 8 to day 14 (week 2) and day 14 until the end (week 3). Recall that the fractal dimension should fall between 1 and 1.5, with a dimension of 1.5 reflecting the fractal self-similarity of an uncorrelated random process. Also recall that higher fractal dimensions are related to lower complexity of the process which generated the sequences. For most patients, the fractal dimensions vary considerably when comparing the 3 consecutive weeks, showing that the complexity of a patient’s activity pattern is not static. The global fractal dimension obtained for the full sequence seems to summarise these weekly dimensions (as one might expect), but it can never do the variation across the 3 weeks justice.

**Table 1:** Various summary statistics for every patient separately. The fractal dimension on a 3-hour scale was obtained for the full activity sequence, and for the three weeks separately, by applying the AAA method with $n_{min} = 1$, $n_{max} = 9\times 60$, $s = 1.1$ and evaluating for $n = 3\times 60$. The mean and standard deviation of the daily sum of activity counts is shown, as well as the correlation between the complexity signal and the activity sequences. This correlation is calculated by sampling the complexity signal (fractal dimension obtained using a 3-day sliding window) three times a day, and comparing these values to the sum of the activity counts contained within the same 3-day windows

Patient ID	Daily activity counts mean $\varvec{\pm }$ std	Fractal dimension (n=3h)				Correlation 3-day activity & fractal dimension
		week 1	week 2	week 3	global	
1	54269 ± 11659	1.293	1.276	1.287	1.287	-0.375
2	102953 ± 13952	1.143	1.217	1.250	1.203	0.039
3	34625 ± 4475	1.219	1.199	1.234	1.222	-0.273
4	75560 ± 13698	1.235	1.259	1.263	1.254	-0.715
5	98137 ± 11050	1.197	1.234	1.283	1.242	-0.179
6	75264 ± 13270	1.227	1.314	1.223	1.251	-0.425
7	74710 ± 13024	1.184	1.181	1.182	1.180	-0.278

To investigate the possible link between personal variations in functioning and complexity of the activity pattern, we report patient-by-patient correlations of the weekly functioning with the weekly fractal dimension. Since we only have indications of high-level functioning on a weekly basis (i.e. the ranking of weeks done by physicians), we could not compare with a more fine-grained temporal evolution of the fractal dimension that can be obtained using the t-AAA method. Table 2 shows the Spearman rank correlation between functioning and complexity. In particular, we compare the ranking of the three weeks in terms of general functioning with the inverted ranking of the weekly fractal dimension (as reported in Table 1). If the reported correlation coefficient is positive, this means that better weeks in terms of functioning are associated with lower fractal dimensions, which are indicative of higher complexity of the activity signal during that week. Since we do not want to compare across patients, we report the correlation on a per-patient basis, meaning each correlation coefficient is calculated only from 3 datapoints (one for each week in the recording). This means that none of the correlations are significant unless there is a perfect alignment between both rankings, as is reflected by their p-values.

**Table 2:** Rank correlations between weekly functioning and weekly activity / weekly fractal dimension. For each patient, the three weeks in the measurement period were ranked in the following way: (1) from worst functioning to best functioning (2) from low complexity (high fractal dimension) to high complexity (low fractal dimension), and (3) from low activity counts to high activity counts. We report the Spearman correlation between rankings (1) and (2) in the first column, and between rankings (1) and (3) in the second column, for each patient separately

Patient ID	Rank corr. functioning & complexity	Rank corr. functioning & activity
1	1 (p < 0.001)	0.5 (p = 0.67)
2	0.866 (p = 0.33)	-0.866 (p = 0.33)
3	0.5 (p = 0.67)	-0.5 (p = 0.67)
4	0.5 (p = 0.67)	0.5 (p = 0.67)
5	-0.5 (p = 0.67)	-1 (p < 0.001)
6	-0.866 (p = 0.33)	-0.866 (p = 0.33)
7	0.5 (p = 0.67)	-0.5 (p = 0.67)

**Fig. 4:**
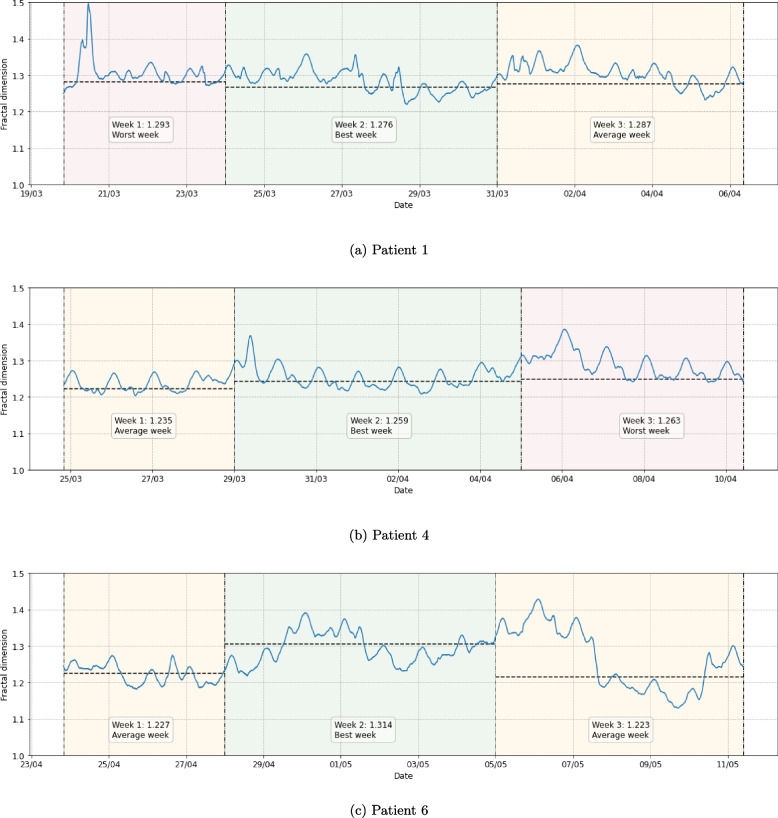
Time-dependent complexity characterisation extracted for a subset of the CFS patients using the t-AAA method. Algorithm 3 was applied to the full activity sequence, with a window width of 3 days (72 hours), a step size of 5 minutes and the following settings of the AAA method: $n_{min} = 1$, $n_{max} = 9\times 60$, $s = 1.1$. The solid (blue) curve depicts the evolution of the fractal dimension on a 3-hour scale (calculated using the slope at $n=3\times 60$). Note that every point on the curve represents the fractal dimension of the past 3 days. The vertical lines split the recording period into 3 weeks, with the first week only spanning 4 days due to the 3-day window which was applied to obtain the fractal dimensions. For comparison with a static approach, the horizontal dashed lines indicate the weekly fractal dimensions as presented in Table 1. Every week is labelled with the value of the static fractal dimension corresponding to that week, as well as its ranking in terms of functioning. This ranking is also reflected in the background colouring of each week, with red, orange and green corresponding to the worst, average and best week, respectively. Similar figures can be found for the other patients in Fig. 5 in the Appendix

**Fig. 5:**
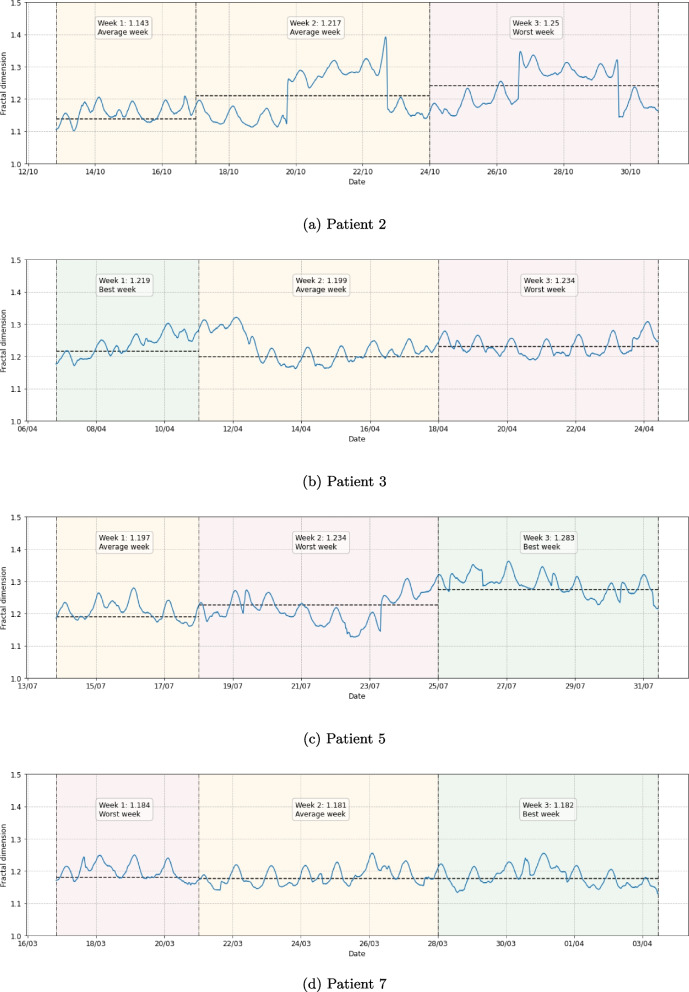
Time-dependent complexity characterisation extracted for the remainder of the CFS patients, using the t-AAA method. See the caption of Fig. 4 for an extended description

The t-AAA method, as described in the Methods and implemented in Algorithm 3, was applied to obtain an evolution of the complexity of each patient’s activity sequence. The solid curve in Fig. 4 depicts the evolution of the fractal dimension over time, for a subset of the CFS patients. We have chosen to focus on this particular subset of patients in the main body of the Results and Discussion, because their time-dependent complexity characterisations have different properties which illustrate the difficulty and heterogeneity of the problem. The same illustrations can be found for the remaining 4 patients in Fig. 5 in the Appendix.

The width of the sliding window is set to 3 days (72 hours). By choosing this width, we strike a balance between the method’s ability to expose higher-level trends (by implicitly averaging out recurring activities, such as sleeping, as well as diminishing the effect of short-term changes in daily schedule, such as weekends) and the desire to detect relevant shorter-term variations in complexity (i.e. in the range of days rather than weeks). To ensure reliability of the allometric aggregation procedure within these 3-day windows, the $n_{max}$ parameter was set to a scale of 9 hours.^3^ As addressed previously, fractal dimensions can (and should) be studied on various scales below this maximum scale setting, but for the current purpose of demonstrating the properties of the time-dependent complexity signal, we focus on a scale of 3 hours.

Figure 4 also contains the static weekly fractal dimensions as reported in Table 1, depicted by the dashed horizontal lines and mentioned in the weekly labels. Finally, indications of the high-level functioning of the patient during each week are illustrated as well, to facilitate the initial exploration of links between functioning and complexity of the activity pattern. Red, orange and green backgrounds respectively correspond to the worst, average and best week in terms of functioning, as reported by the physician who followed up the patient. In the case of patient 6, the physician was only able to indicate week 2 as the best week, meaning the other two weeks are both labelled as average.

### Relation between complexity and activity counts

An important question to ask when evaluating the added value of the time-dependent complexity metric is whether this signal encodes underlying trends which could not be readily extracted from the activity pattern itself. To investigate this, Table 1 presents some general properties of each patient’s activity pattern. We report the average daily activity counts per patient, together with their standard deviation. Daily activity counts are defined as the total sum of all activity counts within a day, and the average is calculated by taking the mean over all 3 weeks in the recording period.

The table also provides an indication on the correlation between the activity patterns and the extracted complexity signal. These correlation coefficients were calculated by sampling the fractal dimensions obtained using a 3-day sliding window (as shown in Fig. 4) three times a day, and correlating these dimensions with the sum of the activity counts contained within each of those segments. The strength of these correlations is measured using Pearson’s correlation coefficient. For all patients, the correlation coefficient is either negative or close to zero.

We also wondered whether weekly levels of activity were related to high-level functioning of each CFS patient. To this end, Table 2 reports the Spearman correlation between the ranking of the three weeks in terms of general functioning (as constructed by the physicians that conducted weekly in-depth interviews with each patient) and the ranking in terms of activity (quantified by the total sum of the activity counts throughout each week in the recording). If the reported correlation coefficient is positive, this means that better weeks in terms of functioning were paired with a higher total of activity counts. Again, for every patient, only 3 datapoints (one per week) could be included in our calculations, explaining the high p-values.

## Discussion

We will now discuss various aspects of our time-dependent complexity method based on the results and figures presented in the previous section. First of all, we argue why a static complexity metric is not sufficient to characterise the observed changes in the fractal dimension of an activity pattern over time. We also explore the following hypothesis: weeks in which a patient showed decreased functioning are coupled with a lower complexity of the week’s activity pattern. Further, we investigate whether the complexity evolution carries any trends which could not be readily extracted from the activity pattern itself and discuss which relations might arise between the two signals and why. Next, we study the extent of scale-dependent variations in fractal dimension and provide an intuitive interpretation of the scaling behaviour. We then compare our method to other similar studies which attempt to characterise the complexity of CFS patients’ activity patterns. Finally, we discuss the limitations of our work and provide guidelines for future work.

### Within-patient variations in complexity over time

Figure 4 illustrates how the fractal dimension of an activity pattern can evolve over time. For some patients, the fractal dimension tends towards both the minimal and maximal theoretical value (respectively equal to 1.0 and 1.5) at different points throughout the recording period. For others, the fractal dimension does not change as drastically, but still seems to evolve over time. We can observe a daily oscillation in the signal, which is more regular for some patients than for others. We refer the interested reader to the supplementary material (see [30]) for a detailed explanation of this phenomenon. In summary, it arises due to an alternation between sleep and wake time. Because we move our sliding window at the small resolution of 5 minutes, these day-night rhythms are present in the outcome of the method. Such momentary oscillations in the fractal dimension are not necessarily informative of longer-term patterns in the behaviour of patients, and when disregarding them we can still observe considerable changes in complexity within the range of days and weeks. From now on, when we talk about temporal changes in complexity, we are referring to these macro-trends, such as the rise and fall of the fractal dimension over multiple days.

While application of the allometric aggregation method to the full activity pattern at once will implicitly average out some more unreliable fluctuations and artefacts, information about the longer-term changes in complexity will be lost as well. Indeed, even though a weekly static fractal dimension seems to (partially) summarise the observed complexity evolution throughout the week, it does not do these variations justice. The time-dependent complexity method allows us to capture how the fractal dimension evolves over a period of several days and weeks. At the same time, studying the general course of this complexity signal can still provide an idea of the overall complexity of the full activity pattern in the long term. From these observations, we learn that a temporal averaging of the fractal dimensions obtained in smaller windows (i.e. the mean of the outcome of the AAA method for all 3-day windows in one week of recording, which would be the average of the t-AAA curve in one week), is not the same as performing the AAA method to get the fractal dimension over one larger window (i.e. the AAA method applied to one week of recording, depicted in the dashed line).

Apart from illustrating the variations in complexity over time, Fig. 4 also reveals the potential of a time-dependent complexity characterisation to capture the personalised nature of CFS. One patient’s complexity signal might behave more erratically than the other (e.g., patient 6 vs. patient 4), reflecting different properties of their activity patterns and possible differences in underlying disease states. Of course, it is not surprising that the properties of the activity patterns differ from patient to patient, as anyone’s daily schedules and events that might interrupt these are entirely different. Apart from this, however, patients can also experience various inhibitions as a result of CFS, which we can expect to leave different marks on the activity pattern and in turn on its fractal properties. Both of these aspects give reason to study the complexity evolution *within* patients, rather than comparing *across* them.

Charts like the ones presented in Fig. 4 could form a useful point for discussion between patients and their clinicians. We imagine the chart could be annotated with further information on patient functioning, important life events, and possibly other temporal signals such as the activity counts and their extracted properties. Even in cases where it proves difficult to identify a mapping from trends in complexity (or other temporal quantifiers anyone might want to investigate) to changes in patient functioning, the ensuing discussion could still enhance the mutual understanding between patient and clinician and provide them with a fresh outlook on the patient’s personal triggers that perpetuate their symptoms.

### Relation between complexity and patient functioning

As outlined in the Background section, we have reason to believe that the evolution of the complexity of the activity pattern of a CFS patient might form a way to quantify changes in their underlying disease state. This forms the motivation to investigate whether changes in complexity (here, measured in terms of fractal dimension) can be related to changes in high-level patient functioning. Prior studies [20, 21] investigated the hypothesis that CFS patients show signs of reduced complexity compared to healthy controls. We propose to move from their idea of comparing a *single* quantification of complexity *between* patients to comparing the complexity over *different windows in time* but *within* a given patient. To this end, we formulate the following hypothesis:

$\mathcal {H}$: *Periods of decreased functioning in CFS patients are associated with a higher fractal dimension (corresponding to a reduced complexity) of the corresponding activity pattern.*

To study this hypothesis, we would ideally compare the time-dependent complexity signal as depicted in Fig. 4 to a fine-grained (i.e. in the order of days) evolution of patient functioning. Currently, we only have a rough ranking of each patient’s three weeks in terms of functioning, meaning we cannot perform such a fine-grained comparison. Instead, we turn to the rank correlations between weekly static complexity and weekly functioning reported in Table 2. While many of these correlation coefficients are positive, which is in line with the hypothesis, the rankings of the weeks in terms of functioning and complexity do not consistently align. For this reason, hypothesis $\mathcal {H}$ has to be rejected based on the data we currently have. Since we do not have a more fine-grained indication of patient functioning on the daily level, we also refrain from speculating about whether sub-weekly trends in complexity (e.g. the rise and fall of the fractal dimension within a single week, which often occurs in Fig. 4) could possibly be related to changes in the patient’s functioning during that week.

From Table 2 it is also clear that the weekly activity counts mostly show a negative correlation with weekly functioning, though again both rankings are not consistently aligned. This shows that the activity pattern in its simplest form is not highly indicative of patient functioning either.

### Relation between physical activity and complexity

We can ask ourselves whether the time-dependent complexity signal encodes any trends which could not readily be extracted from the activity pattern itself. This question can be partially answered by studying the linear relation between the two signals. As illustrated by the last column in Table 1, the linear correlation coefficient obtained for samples extracted from both signals varies across patients. For some patients, the activity counts within a window are strongly correlated with the fractal dimension obtained for that part of the activity sequence, while for others this correlation is much less strong. For patients of the former type (for example, patient 4), the time-dependent complexity method might show less potential for revealing novel properties of the activity pattern, again emphasising the personalised care framework in which this method should be viewed.

Whether weak or strong, the correlations which are not close to zero are all negative. This means that segments with a higher total of activity counts are usually paired with lower fractal dimensions, suggesting the presence of stronger fractal correlations (generated by a more regular process) in these sequences than for their less active counterparts. Indeed, if there is a higher total activity in the studied 3-day segment, the variation of the aggregated activity counts signal (i.e., when taking subsequent steps in the allometric aggregation procedure) is expected to increase more rapidly relative to the total activity, compared to segments with a lower total activity. This results in a steeper slope on the log-log plot and a lower fractal dimension. Intuitively, we can interpret this as the fractal organisation of movement being less similar to that of a random process when it is part of a more physically intense activity. We hypothesise that such activities are, on average, executed with more purpose and structure than a sedentary activity which is interrupted by more randomly dispersed movements.

At the same time, there are several instances where higher activity counts do not result in higher complexity (otherwise the correlation coefficients in Table 1 would be much closer to -1). While investigating the activity signals in detail, we realised that relatively short-term high peaks in activity counts can interrupt the more regular organisation of the activity sequence. This then seems to lead to a momentary increase of the fractal dimension, contradicting the general observed trend of activity sequences with higher counts being paired with lower fractal dimensions. Since patients indicated the type of activities undertaken throughout the day in the daily surveys, we were usually able to identify these momentary increases in activity counts as intensive sports, such as fitness or running. Indeed, as these relatively constrained movements bear less relation to the surrounding free-range movements, the assessment of such a sequence’s complexity across timescales will be more similar to that of an uncorrelated random process (which, in theory, has a fractal dimension of 1.5).

In order to leverage the potential of the time-dependent complexity method to reveal novel properties of patients’ activity patterns, insight into which aspects of the activity pattern directly influence the obtained fractal dimension is needed. It might be appropriate to remove or reduce large momentary peaks in activity counts^4^ before calculating the fractal dimension, as their influence might distort the obtained complexity disproportionately. In this way, the obtained fractal dimensions would disregard extreme interruptions in activity counts, and the signal might be more reflective of the fractal correlations present in the free-range activity and movements of the patients. The part of the remaining trend which can be attributed to the described negative linear correlation between activity counts and the fractal dimension could then be removed, leaving only the part of the observed trend which reveals fractal properties of the activity signal which were not obvious from the activity pattern itself. We would expect this latter part to be more reflective of underlying changes in the regulation of the patient’s complex adaptive system, and exposing it could allow the identification of stronger relations with patient functioning and their general disease state.

### Scale-dependent variations in complexity

The goal of this section is to dig deeper into the properties of the AAA method that are related to variations across scales. We both aim to provide intuitive explanations for some phenomena observed in the scaling behaviour of the activity sequences we studied, as well as motivate some of our choices regarding the parameters of the (t-)AAA method that were used to obtain the results reported in previous sections.

Apart from its variation across time, we have also shown that the fractal dimension can vary according to the scale parameter (represented by *n*, the level of aggregation in the allometric aggregation algorithm). Of course, as the complexity metric is designed to capture the fractal correlations across scales, the slope obtained around a particular value of the scaling parameter *n* will partially depend on the strength of the correlations on all scales, from $n_{min}$ to $n_{max}$. It is the overall coherence of the variations within various scales, ranging from a level of minutes to a level of hours, that decides whether the time series shows any sense of self-similarity and to what extent. However, at the same time, the lower range of scales may still exhibit a different strength of fractal correlations than the higher range of scales, which is illustrated in Fig. 2: the relation between mean and variance is better captured by a polynomial than by a single straight line. In this case, we see that lower scales exhibit steeper slopes, corresponding to lower fractal dimensions. The discussion that follows links the inner workings of the allometric aggregation method to an intuitive explanation for this observation.

Imagine dealing with a time series generated by a random uncorrelated process, characterised by a certain mean and variance. When aggregating the samples into blocks of size *n*, we essentially take the sum of *n* independent and identically distributed variables with identical mean and variance. Basic statistics allow obtaining the mean and variance of the resulting aggregated time series as the original mean and variance both multiplied by *n*, respectively. On a log-log scale, this results in a slope of 1 (and a fractal dimension of 1.5), since both mean and variance increase with the same amount. When we are dealing with a process that exhibits some regularity, consecutively generated samples will not be independent anymore. As we aggregate the series into blocks of *n* samples, the mean will still increase with factor *n*, but now the variance will increase with an additional amount proportional to the covariance between the consecutive samples (reflecting the strength of the dependence between the samples generated by the process). On a log-log scale, this results in a slope that is steeper than 1. This translates into a fractal dimension lower than 1.5, reflecting that there is some regularity in the signal, as is indeed the case for the activity patterns we present here. Since the slope in the lower scaling ranges is steeper than in the higher ranges, we can state that the signal shows more regularity for these lower scales. Our intuition would indeed confirm that there should be more regularity in movements when these are compared from one minute to the next, as particular activities often span a time-frame larger than a couple of minutes.

At higher scales, the slope becomes lower, resulting in a higher fractal dimension. Suppose, for example, that we are considering 3-hour scales. This effectively means that we are comparing consecutive 3-hour segments and calculating their variation in terms of total aggregated activity counts enclosed in these segments. We can expect that the properties of this time series correspond more to those of a random signal than was the case for lower scales, as we may be comparing across activities less related to one another. However, the fact that the slope of the mean-variance relation still remains higher than one shows that some regularity is still present in the signal at this scale. There is usually still some purpose behind a person moving from activity to activity, explaining why activity patterns show a degree of self-similarity even on higher timescales.

The question that follows is which range of scales is more important when analysing the complexity of our activity patterns. We can argue that scales of a different order of magnitude each capture a different notion of complexity. The way the strength of the mean-variance relation changes across scales as we move to higher ranges also contains information about the complexity of a time series. When we discard information on the self-similarity of the signal when viewing it on lower scales, we discard information about how the subject’s movement is hindered when operating within a specific activity. On the other hand, when discarding the fractal dimension obtained for higher scales, we discard indications of the activity pattern’s self-similarity across higher-level tasks and daily schedules. In the current work, we have made the decision to focus on the fractal dimension obtained for higher scales ($\sim$ 3 hours) when presenting our time-dependent complexity characterisation, in order to simplify the presentation and analysis of these results. However, future work should focus on developing a temporal complexity representation which also contains information about the signal’s scale-dependent behaviour, without disregarding the fractal properties in the lower range of scales ($\sim$ minutes).

Apart from taking into account these considerations on the changes in fractal dimension across scales, there is also a trade-off to consider when choosing the width of the window used to obtain the time-dependent complexity signal. The slope of the mean-variance relation can only be calculated reliably if enough segments are created in the higher-level aggregation steps. This induces a limitation on the width of the window which selects the part of the activity series to feed into the algorithm. For example, when we aim to study the fractal correlations up to a scale of 3 hours, the window should not become much smaller than 24 hours. While a larger window allows for more reliable estimates, it also results in a slower manifestation of the changes in the activity series’ properties. Intuitively, it is not always clear which choice of window and scale is most appropriate in light of this trade-off. From experience, we recommend the window size to be at least 8 times as large as the maximum scale ($n_{max}$) one desires to study. The step size, which we fixed to 5 minutes, can be chosen freely according to the desired granularity of the complexity signal, although it will impact the execution time.

The 3-day sliding window we chose to apply is sufficiently large in relation to the 3-hour scale we focus on, which would require a 24-hour window at least. Going for a larger window increases the reliability of the estimated means and variances in the AAA algorithm, reducing the amount of sharp momentary peaks in the complexity signal which would make it harder to extract and interpret a global trend in complexity. A larger window also decreases the magnitude of the daily oscillations which arise due to an alternation between sleep and wake time, a phenomenon we have pointed out briefly when discussing the within-patient variations in complexity. Furthermore, we believe this window size better reflects the level at which we desire to observe changes in complexity for these patients. We do not want momentary changes in the behaviour of the patients, which may not bear a relation to changes in the manifestation of their illness, to have an immediate impact on the complexity characterisation. Rather, we want to observe slower but more permanent changes in complexity, which might still be triggered by a singular event in the patient’s timeline, but which bear a more long-lasting impact. We believe that a window of 3 days is long enough to average out the effect of irrelevant momentary changes, while the resolution of the information contained in the resulting complexity signal is still high enough compared to its full length of 3 weeks.

### Comparison with other studies

We identified only two other studies which investigated the complexity of CFS patients’ activity patterns in particular. Ohashi et al. [20] used two techniques, detrended fluctuation analysis (DFA) and wavelet transform modulus maxima (WTMM), to estimate the fractal scaling exponent of 14-day physical activity time series. They reported that CFS patients’ activity patterns showed indications of reduced complexity compared to healthy controls. Burton et al. [21] reported similar observations, using the allometric aggregation method (cf. Algorithm 1) to estimate the fractal dimension of 12-day activity patterns. What both studies have in common, is the fact that they characterised the fractal properties of the 2-week long time series using a single static complexity value per patient, as opposed to the time-dependent characterisation presented here. Both studies were only able to significantly discriminate very inactive CFS patients from healthy controls based on the fractal properties of their activity time series.

As motivated previously, we decided to use the same metric as Burton et al. [21] to allow for a closer comparison. Furthermore, we followed Burton et al. [21] in converting the raw acceleration recordings to activity counts. Our observations suggest that their results should be interpreted with caution. Table 1 illustrates how the fractal dimensions obtained for the first, second and third week of the recording are relatively far apart for some patients. Some variations are around the same order of magnitude as the differences reported by Burton et al. [21] between the average fractal dimension obtained for their control group (1.14), their active CFS patient group (1.16) and their pervasively inactive CFS patient group^5^ (1.21) (though the scale for which these dimensions were obtained is unclear). This indicates a lack of robustness of the static allometric aggregation method, especially for its application in a diagnostic context: the fractal dimension of a week-long activity pattern might classify a subject to fall within the CFS patient group one week, but not the next.

The observed temporal variations in complexity might partially explain why little statistical difference was found between the fractal dimension of active CFS patients and controls. As opposed to the method applied by Burton et al. [21], a time-dependent complexity characterisation of the activity pattern over multiple weeks does not discard information on the variations in fractal dimension. While identifying new methods for diagnosis of CFS is not the focus of the current work, we can argue that retaining this information might lead to a more robust complexity-based diagnostic method.

We can also question whether comparison between groups of patients and controls, based on fractal dimensions obtained for a particular scale, is warranted. Burton et al. [21] did not provide a motivation for the scale upon which they evaluated the fractal dimension, and it is unclear whether multiple scales were tried and how their results would differ in these cases. In any case, Fig. 3 illustrates how comparisons between patients can differ when the fractal correlation strength in different scaling ranges is explored. The same might be the case if we compared these patients with healthy controls: a patient might have a lower fractal dimension on a scale of 30 minutes but a higher one on a scale of 3 hours. It is certainly necessary to stick to one particular setting for the scale parameter to ensure comparability of fractal dimensions (as was done by Burton et al. [21]), but apart from this it would also be advised to explore various scale settings and report whether this impacts the significance of the results.

Both Ohashi et al. [20] and Burton et al. [21] only reported significant differences for so-called pervasively inactive CFS patients when comparing them to a group of healthy controls. Patients who are relatively active despite their CFS diagnosis could not easily be identified. The activity patterns for the inactive patient group are likely very static (showing permanently low activity counts), and in this case it makes sense that the fractal dimension obtained for such activity patterns would be relatively static as well, allowing them to be discriminated from the group of healthy subjects. Additionally, in the case of these pervasively inactive patients, we see that Burton et al. [21] were initially able to identify a significant difference in mean activity counts between this group of CFS patients and their matched healthy counterparts. It begs the question whether a complexity-based separation of these groups is even needed, if the activity pattern in itself already suffices for this purpose. Though we cannot directly compare the patients included in our trial to theirs, based on the interviews and objective activity counts, we would not classify any of the patients included in our pilot trial as pervasively inactive. It is exactly for this rather active group of patients that we expect an evolution of the complexity to be more informative than a single static complexity metric.

### Limitations and future work

We have proposed an extension of the allometric aggregation method to capture the evolution of complexity over time. While we applied it to activity sequences in the context of CFS patients, the t-AAA method is sufficiently general to be applied to any time signal obtained in- or outside a clinical context. Future work could explore its potential to extract temporal variations in complexity from other time series. For example, as fractal properties of HRV signals have been shown to contain information which can be linked to heart failure [35], we can expect quantification of variations in the complexity of the HRV signal to be useful in contexts outside of CFS as well.

Apart from showing that the fractal dimension evolves over time, we also showed that it varies across scales: the fractal properties are different when evaluated around scales at the level of minutes vs. hours. We made a choice to focus on the evolution of the complexity on a 3-hour scale, but the fractal behaviour of the activity pattern in the lower scale range is different, and its variation over time likely contains complementary information. In the future, we should try to find a representation of the complexity and its variation over time which takes this scale-dependent behaviour into account as well.

Furthermore, additional insight is needed into the relationship between certain properties of the activity pattern and the influence these have on the fractal dimension which is derived from it. In our comparison of complexity with activity, we discussed the possible distortion of complexity as a result of large momentary peaks in activity counts (which might occur when a patient engages in heavy exercise). We also described the observed overall negative linear correlation between activity counts and the fractal dimension. Given these observations, we propose that future research should jointly model complexity and activity to account for the relation between both.

Seeing as we were unable to confirm our hypothesis that periods of decreased functioning are related to a decrease in complexity of the CFS patient’s activity pattern, it seems unlikely that the proposed complexity metric (applied to physiological outputs such as activity) can adequately quantify a patient’s state of well-being. We foresee that future research should investigate the potential of a multi-modal approach, where various metrics that can be continuously tracked (e.g. activity counts and derived features, possibly including complexity) are combined into one monitoring system. Instead of focusing on a predefined metric, such as the method of allometric aggregation, we should look into machine learning-based methods to obtain a personalised temporal representation of each patient, which can serve as a quantification of the underlying properties of the patient’s CAS.

Finally, it would be useful to repeat the conducted analysis for a larger and more diverse group of patients, e.g. including patients who have only recently received a diagnosis and have not yet developed many coping mechanisms. The purpose of this extension would not be to compare results across patients, but rather to have a broader view on the extent of within-patient variations in complexity and functioning. We pose that such a follow-up study should collect more fine-grained (i.e. on the daily level) indications of general functioning. This would allow a more direct comparison with any fine-grained temporal quantifiers derived from the activity pattern, facilitating the development of multi-modal temporal representations that are predictive of the personal functioning.

## Conclusions

Past studies have shed light on the potential of complexity metrics (extracted from physiological time series like activity patterns and HRV measurements) as a quantification of the underlying disease state of CFS patients, though much additional research is needed into the robustness of such methods. In our work, we focus on investigating the temporal changes in complexity, which the classical static methods cannot capture. To this end, we have presented a method which can quantify the variations in fractal dimension of a physiological signal over time, by extending the method of allometric aggregation. We applied our new time-dependent method to characterise the fractal correlations of physical activity patterns and compared the outcome with the original static approach. This illustrated the extent of within-patient variations in complexity, which were significant and justify the need for such a time-dependent characterisation. Given the time-varying nature of complexity that is revealed using our method, we are forced to reconsider the utility of a previous study by Burton et al. [21] that characterises long-term activity signals using a single static complexity value. Although the authors did not explicitly state the fractal dimension alone could be a diagnostic tool (due to the non-significant difference between patients and controls in terms of fractal dimension) they did suggest its potential value as an objective CFS indicator. Based on the results of our within-patient experiments, we argue this is highly unlikely.

Apart from presenting a way to capture variations in complexity over time, we also explored the possible link between personal variations in functioning and the complexity of a patient’s activity pattern. We tested this by comparing each patient’s high-level weekly indications of patient functioning with their weekly measures of complexity. Based on this, we could not confirm our hypothesis, which stated that weeks during which a patient showed decreased functioning were expected to be coupled with a lower complexity of the activity pattern. For this reason, we conclude that complexity alone cannot sufficiently capture latent changes in functioning. Instead, we should move towards building systems that jointly model multiple observable features and their extracted metrics (among which the fractal dimension could be one of many), in order to accurately track patient well-being using objective continuous measurements. To accommodate future work on this front, we have published our novel dataset which aligns continuous activity recordings (3 weeks) from 7 CFS patients with daily indications of symptom severity, perceived physical activity, stress levels, etc. The data, which is freely available at our Github repository [30], can be used to explore patient-specific triggers for dysfunctioning and their relation to physical activity or derived metrics.

While we do not show a conclusive use for the time-dependent complexity metric in the CFS use-case, the method itself can be readily applied in other settings, be it other physiological signals (e.g. heart-rate variability), in the context of other clinical disorders that could benefit from the complex adaptive systems way of thinking, or even outside of the medical domain. To the best of our knowledge, the current work is the first to present a method which can characterise the evolution of the complexity over time, rather than representing the complexity of an ever-changing physiological signal by one single, static complexity value.

## Figures and Tables

**Figure:**
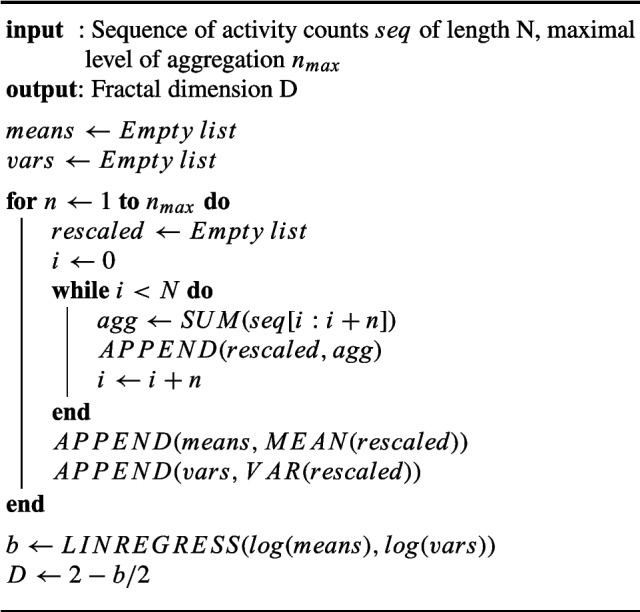
**Algorithm 1** The original allometric aggregation algorithm as defined by West [8]

**Figure:**
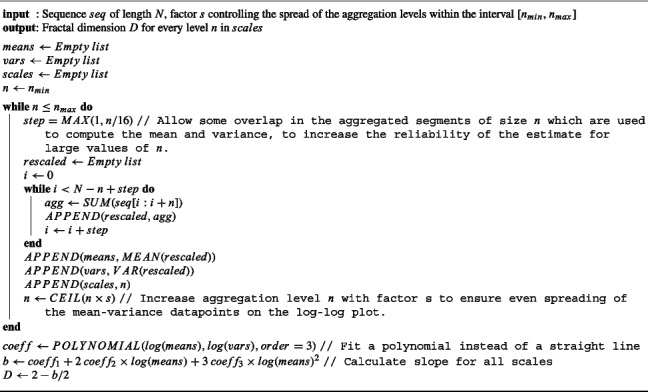
**Algorithm 2** Adapted allometric aggregation (AAA) method to calculate the fractal dimension of a time series. The comments in the pseudocode draw attention to the changes which were made compared to Algorithm 1

**Figure:**
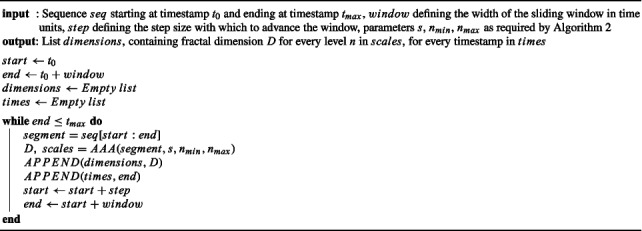
**Algorithm 3** The time-dependent adapted allometric aggregation (t-AAA) method which extracts a list of fractal dimensions, simultaneously over time and across various scales. A time window slides across the activity sequence and calculates the fractal dimension (for various scales) for the activity segment contained within that particular window. The algorithm makes use of the adapted allometric aggregation method (indicated by *AAA*) as defined in Algorithm 2

## Data Availability

The dataset analysed in the current study is available in our Github repository: https://github.com/prabaey/time-dependent-complexity (data folder). The data consist of preprocessed accelerometer recordings (in the form of activity counts sequences), daily responses to surveys and weekly indications of general functioning per patient. The raw accelerometer data are not publicly available due to the large volume of these long-term recordings, but are available from the corresponding author on request.

## Appendix

In the Methods section, we introduced three algorithms that aim to capture the complexity of a time series, either through a static fractal dimension or dynamically over time. Algorithm 1 implements the original allometric aggregation algorithm as defined by West [8]. We introduced a couple of changes in the original algorithm, to address certain issues with its practical application and to allow it to capture the fractal dimension across various scales. Our implementation of the adapted version of the allometric aggregation method (referred to as the AAA method) is presented in Algorithm 2, which also points out where these adaptations were made. Finally, Algorithm 3 implements the time-dependent complexity method, which applies the AAA method to obtain the fractal dimension within consecutive windows. We publish our Python implementation of these algorithms in the supplementary material (see [30]). There, we also include a comprehensive explanation of the algorithm parameters, and lay out the changes we made to the original allometric aggregation method in depth. These in-depth explanations are meant for anyone who would like to reuse our method or reproduce our results.

Figure 5 presents the complexity evolution for patients 2, 3, 5 and 7 (i.e., those not shown in Fig. 4), together with their static fractal dimensions for each week and the ranking of their functioning. These figures are merely presented to the reader as additional examples of the application of our method, since we did not discuss these patients’ use-cases in depth.

## Abbreviations

AAA: Adapted Allometric Aggregation
t-AAA: temporal Adapted Allometric Aggregation
BDS: Bodily Distress Syndrome
CAS: Complex Adaptive System
CFS: Chronic Fatigue Syndrome
HRV: Heart-Rate Variability

## Footnotes

1 Please note that we did not intend to reproduce their study, nor confirm or reject their results, which is why we did not include a large pool of CFS patients and had no control group. Rather, by studying the within-person variations in complexity, we want to shed a new light on the non-static nature of the complexity metric they used.

2 Raw accelerations are still available for further analysis and can be requested along with the published counts sequences.

3 The ratio between the maximal scale $n_{max}$ and the total length of the activity sequence used as an input to the AAA method should be sufficiently large. If not, too little samples are obtained after aggregating up to a higher scale, rendering the estimation of the mean and variance of the rescaled time series unreliable.

4 We should be careful to only remove such peaks if they can be certainly linked to a specific high-intensity activity purposefully undertaken by the subject. This information can potentially be extracted from daily surveys filled in by patients or by markers left in the activity data (for example, by requesting patients to clap 5 times before starting a sporting activity). Furthermore, we should not disregard these events entirely, even if they are not included in the calculation of the complexity signal. They should still be kept in mind while studying their repercussions in terms of symptoms and general functioning in the days that follow, and how those aspects might be reflected in the complexity signal.

5 CFS patients were categorised as pervasively inactive if their daily activity counts were smaller than the average daily counts (based on the entire group of CFS patients) for 90% of all days in the recording period.
